# How Covid-19 changed the epidemiology of febrile urinary tract infections in children in the emergency department during the first outbreak

**DOI:** 10.1186/s12887-022-03516-7

**Published:** 2022-09-15

**Authors:** Laura Cesca, Ester Conversano, Federica Alessandra Vianello, Laura Martelli, Chiara Gualeni, Francesca Bassani, Milena Brugnara, Giulia Rubin, Mattia Parolin, Mauro Anselmi, Mara Marchiori, Gianluca Vergine, Elisabetta Miorin, Enrico Vidal, Cristina Milocco, Cecilia Orsi, Giuseppe Puccio, Licia Peruzzi, Giovanni Montini, Roberto Dall’Amico, on the behalf of the Italian Society of Pediatric Nephrology (SINePe)

**Affiliations:** 1 Pediatria - Ospedale Civile S. Maria Degli Angeli Di Pordenone, Pordenone, Italy; 2 Pediatric Department, Institute for Maternal and Child Health - IRCCS “Burlo Garofolo”, Trieste, Italy; 3 Pediatric Nephrology, Dialysis and Transplant Unit, Fondazione Ca’ Granda IRCCS, Policlinico Di Milano, Milano, Italy; 4 Dipartimento Di Pediatria, Ospedale Papa Giovanni XXIII, Bergamo, Italy; 5 Clinica Pediatrica Degli Spedali Civili Di Brescia, Brescia, Italy; 6 Unita’ Operativa Complessa Di Pediatria, Ospedale Regionale Di Bolzano, Bolzano, Italy; 7 Pediatria, Ospedale Universitario Della Donna E del Bambino Di Verona, Verona, Italy; 8 Unità Operativa Complessa Di Pediatria, Dipartimento Strutturale Materno-Infantile, Ospedale San Bortolo, Vicenza, Italy; 9 Pediatric Nephrology, Dialysis and Transplant Unit, Department of Women’s and Children’s Health, University-Hospital, Padua, Italy; 10 Unità Operativa Complessa Di Pediatria Dolo-Mirano, Dolo, Italy; 11 Unità Operativa Complessa Di Pediatria E Patologia Neonatale, Ospedale Dell’Angelo Di Mestre, Mestre, Italy; 12 Department of Pediatrics, Rimini Infermi Hospital, Rimini, Italy; 13 Struttura Complessa Di Pediatria, Ospedale Civile Di Latisana-Palmanova, Latisana, Italy; 14 Division of Pediatrics, Department of Medicine (DAME), University-Hospital of Udine, Udine, Italy; 15 Divisione Di Struttura Operativa Complessa Di Pediatria, Ospedale San Polo, Monfalcone, Italy; 16 Nefrologia Pediatrica - Ospedale Regina Margherita - Città Della Salute E Della Scienza Di Torino, Torino, Italy; 17 Giuliana and Bernardo Caprotti Chair of Pediatrics, Department of Clinical Sciences and Community Health, University of Milano, Milano, Italy

**Keywords:** Urinary tract infection, Covid19, Diagnosis delay

## Abstract

**Background:**

The first Covid-19 pandemic affected the epidemiology of several diseases. A general reduction in the emergency department (ED) accesses was observed during this period, both in adult and pediatric contexts.

**Methods:**

This retrospective study was conducted on the behalf of the Italian Society of Pediatric Nephrology (SINePe) in 17 Italian pediatric EDs in March and April 2020, comparing them with data from the same periods in 2018 and 2019. The total number of pediatric (age 0–18 years) ED visits, the number of febrile urinary tract infection (UTI) diagnoses, and clinical and laboratory parameters were retrospectively collected.

**Results:**

The total number of febrile UTI diagnoses was 339 (73 in 2020, 140 in 2019, and 126 in 2018). During the first Covid-19 pandemic, the total number of ED visits decreased by 75.1%, the total number of febrile UTI diagnoses by 45.1%, with an increase in the UTI diagnosis rate (+ 121.7%). The data collected revealed an increased rate of patients with two or more days of fever before admission (*p* = 0.02), a significant increase in hospitalization rate (+ 17.5%, *p* = 0.008) and also in values of C reactive protein (CRP) (*p* = 0.006). In 2020, intravenous antibiotics use was significantly higher than in 2018 and 2019 (+ 15%, *p* = 0.025). Urine cultures showed higher *Pseudomonas aeruginosa* and *Enterococcus faecalis* percentages and lower rates of *Escherichia coli* (*p* = 0.02).

**Conclusions:**

The first wave of the Covid-19 pandemic had an essential impact on managing febrile UTIs in the ED, causing an absolute reduction of cases referring to the ED but with higher clinical severity. Children with febrile UTI were more severely ill than the previous two years, probably due to delayed access caused by the fear of potential hospital-acquired Sars-Cov-2 infection. The possible increase in consequent kidney scarring in this population should be considered.

## Introduction

UTI represents one of the most common bacterial infections in children and a frequent cause for evaluation in the ED. In the USA, the prevalence of UTI diagnosis in pediatric ED visits is about 0,43% [1]. The highest incidence rate is reported during the first year of life, without significant differences between the genders [2, 3].

A high percentage of children with UTIs is diagnosed in the ED. A study conducted in the USA between 2006 and 2011 reported that 1.904.379 children aged ≤ 17 years were evaluated in ED for a first UTI. While most of these patients were discharged after starting treatment, 4.7% were hospitalized [1]. This data reflects the content of many international guidelines regarding UTI, which recommend hospitalization only in specific clinical conditions [4–7]. Over the last few years, several American investigators have noticed an increasing number of pediatric ED visits, and, meanwhile, the number of UTIs diagnosed in the ED has also risen [1, 8, 9].

In November 2019, the first cases of Coronavirus disease 19 (Covid-19) were declared in Wuhan (China), and severe acute respiratory syndrome Coronavirus 2 (Sars-Cov-2) spread throughout the world, causing more than 2 million deaths [10, 11] at the time of study data collection. Sars-Cov-2 spread across the Italian peninsula somewhat unevenly, with significant differences in viral spread detected between the various regions [12].

During the first Covid-19 pandemic, pediatric ED visits were reduced in the Italian [13, 14] and global context [15, 16]. Several reports demonstrated how the Covid-19 pandemic influenced the epidemiology of many adult conditions [17, 18].

A possible explanation for this decrease lies in the fewer cases of traumatic injuries and infectious diseases observed in the ED due to the limitations imposed by the government to limit Sars-Cov-2 spreading and the reluctance of parents to bring their children to the ED [19, 20].

We assumed that also fewer UTIs were diagnosed in the ED during the first Covid-19 outbreak because the proportion of cases less severe was treated at home rather than brought to the attention of ED. We investigated the characteristics of febrile UTIs diagnosed in a cohort of Italian EDs during March–April 2020 and compared them with those of the previous two years, to assess the diagnosis rate of febrile UTI during the first Covid-19 pandemics compared to the two previous years.

## Methods

### Study setting and design

We conducted a retrospective multicenter study to analyze the influence of the Covid-19 pandemic on the diagnosis of febrile UTIs in the pediatric ED setting.

#### Patients

The study population consisted of children (0–18 years) diagnosed with a febrile UTI in the ED, in March and April 2020, during the first peek of the Covid-19 pandemic in Italy and the same months of the two previous years (2018–2019).

#### Baseline data

We collected data from the ED database. For each visit to the ED, the database registered age and gender of the patient, date of the visit, nursing triage category, access mode (spontaneous/voluntary or referral by a health care provider), history of previous UTI or congenital anomalies of the kidney and urinary tract (CAKUT), days of fever before and after hospital admission, hospitalization and days of hospitalization discharge diagnosis, medical recommendations at discharge, antibiotics prescribed, and administration, results of renal and bladder ultrasound, when performed.

According to the Italian Society of Pediatric Nephrology (SINEPE) guidelines, blood samples for laboratory investigations were collected if the child was hospitalized or was less than three months old. Upon arrival at the emergency room, blood samples were taken. Laboratory data were collected at the admission, including white blood cell count (WBC, cell/ul), C-reactive protein (CRP, mg/dl), procalcitonin (ng/ml), blood culture, urine collection methods (sterile bag, clean-catch midstream urine specimen, or catheterization), urine dipstick and urine culture, antibiotic resistance profile of the isolated bacterium. According to SINEPE guidelines, UTI was characterized by the presence of fever (≥ 38 °C) and a positive urine dipstick (leukocytes and/or nitrites) confirmed by urine culture, defined as the presence of a single bacterium in urine culture, > 10.000 CFU/mL in a urine sample from bladder catheterization, > 50.000 CFU/mL in clean voided urine, and > 100.000 CFU/mL from the bag. Patients with more than one ED access for UTI or with negative or polymicrobial urine culture were excluded. Moreover, the total number of ED admissions of pediatric patients during March–April for the same years was recorded. The study’s primary outcome was to evaluate the diagnosis rate of febrile UTI during the first Covid-19 pandemic compared to the two previous years. The secondary outcome was to assess the indicators of different clinical severity of febrile UTIs. The SINEPE group supported the study, and the study was conducted on the behalf of the Italian Society of Pediatric Nephrology (SINePe). This study was approved by the Institutional Review Board of the Azienda Sanitaria Friuli Occidentale-Pordenone, Italy.

#### Outcomes

The study’s leading outcome was to evaluate the diagnosis rate of febrile UTI during the first Covid-19 pandemic compared to the two previous years. The secondary outcome was to assess the indicators of different clinical severity of febrile UTIs.

### Statistical analysis

Statistical analysis was conducted using the open-source software R: R Core Team (2021). R: A language and environment for statistical computing. R Foundation for Statistical Computing, Vienna, Austria. URL https://www.R-project.org/. For the analysis of unidimensional data (such as counts of ED visits and UTI diagnoses in the three years considered), a uniform distribution in the three years was assumed as a null hypothesis, and a chi-squared test for goodness of fit was performed versus the assumed distribution. A Chi-Square test for independence was employed to analyze the association between two categorical variables. Almost all the numeric variables we considered had a non-normal distribution. Therefore, they were summarized as median, IQR and range, and non-parametric tests (Wilcoxon, Kruskal–Wallis tests for independent groups) were utilized to compare their distribution in two or more independent groups. A Wilcoxon test for paired groups was used to analyze the distribution of per cent variations in the participating centres. *P* values lower than 0.05 were considered significant.

## Results

Seventeen Italian general and pediatric EDs participated in the study (see appendix 1), with 128.079 ED visits and 339 diagnoses of febrile UTI recorded.

Counts of ED visits and UTI diagnoses were rather stable in the two pre-pandemic years: ED visits were 57,909 in March–April 2018 and 55,965 in March–April 2019, while UTI diagnoses were 126 in March–April 2018 and 140 in March–April 2019. Both counts dropped dramatically in 2020. Therefore, we considered for the analysis only two periods:

- Period 1: the two pre-pandemic years (2018 and 2019), March and April
- Period 2: the pandemic year (2020), March and April

Of course, for period 1 absolute counts (ED visits and UTI diagnoses) were expressed as mean per year, to be comparable to absolute counts of the second period. Values for the two periods are shown in Table 1. Baseline and demographic characteristics of the study population of children with febrile UTI diagnosis are summarized in Tables 2 and 3.

**Table 1:** Counts of ED visits and UTI diagnoses, and rate of UTI diagnoses (UTI diagnoses / ED visits) in the two periods

	Period 1	Period 2	*p* value
Total number of ED visits	56,937 (mean per year)	14,195	< 0.001
Total number of UTI diagnoses	133 (mean per year)	73	< 0.001
Total rate of UTI diagnoses	0.23%	0.51%	< 0.001

**Table 2:** Children with UTI diagnosis: baseline data and data collected after admission to ED in the two periods: categorical data

	Period 1	Period 2	*p* value
Gender (Female)	53.4%	42.5%	0.10
Access mode (referred by pediatrician)	16.2%	21.4%	0.31
Previous UTI	23.7%	23.6%	0.99
Previous urinary tract malformations	20.2%	26.8%	0.23
Hospitalization	43.5%	63.0%	0.008

**Table 3:** Children with UTI diagnosis: baseline data and data collected after admission to ED in the two periods: continuous data

	Period 1	Period 2	
	Median	Median	*p* value
Age (years)	0.8	0.6	0.97
WBC 10^3/μL	15.2	17.0	0.32
N 10^3/μL	8.7	10.0	0.35
CRP (mg/dL)	5.8	8.6	0.006

The total number of ED visits was remarkably lower in period 2 compared to period 1 (14,195 vs a mean of 56,937, -75.1%). The number of UTI diagnoses in period 2 was also significantly lower than in period 1, but the decrease was smaller than the one related to ED visits (73 vs a mean of 133, -45.1%). Consequently, the rate of UTI diagnoses showed a statistically significant increase in period 2 (0.51% vs 0.23% in period 1, + 121.7%).

**Fig. 1:**
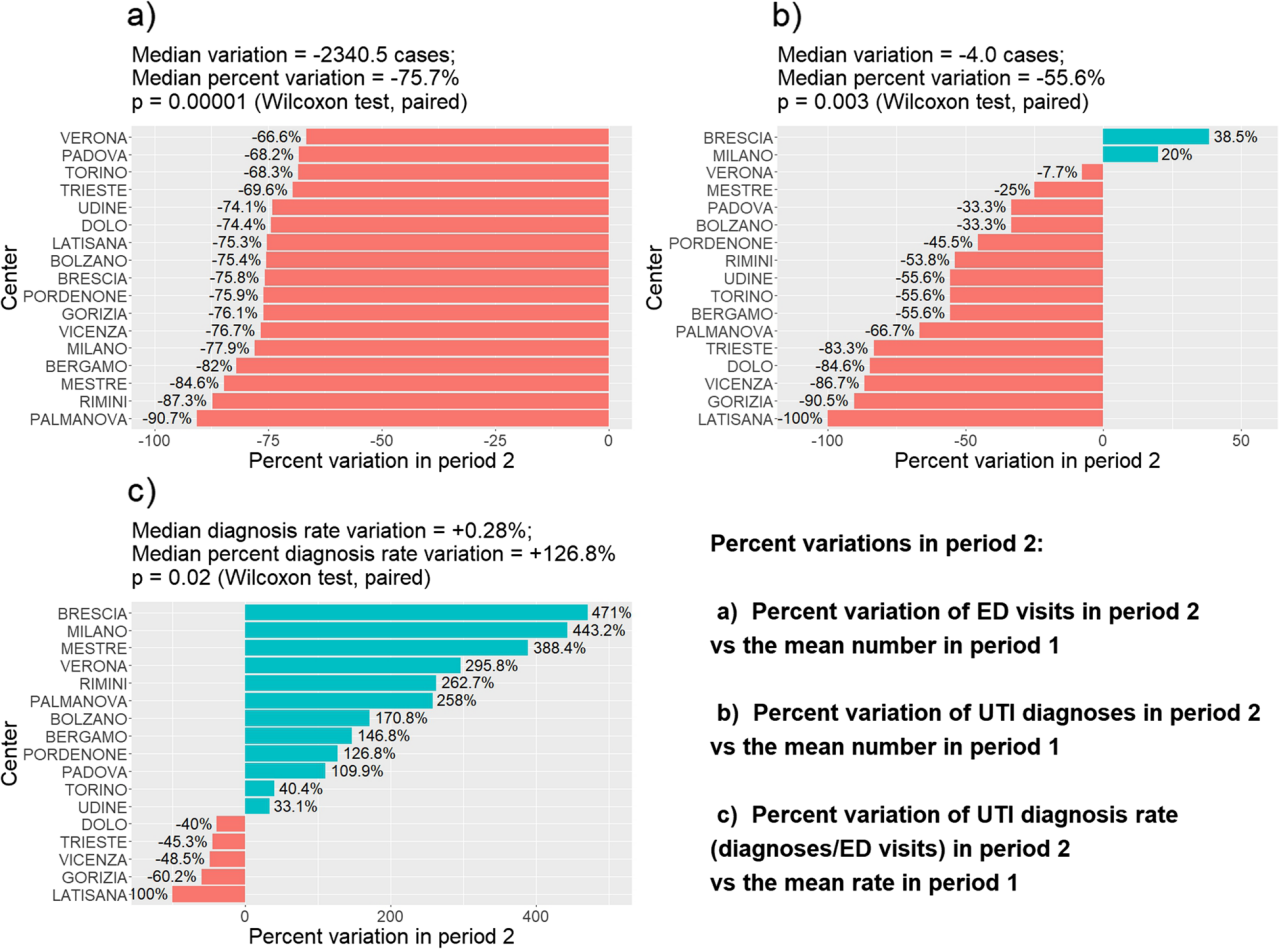
Distribution of per cent variation between period 2 and 1 in the absolute number of ED visits (**a**), the absolute number of UTI diagnoses (**b**), and UTI diagnosis rate (**c**) between the centres participating in the study. A paired analysis was performed to detect the general trend prevailing in centres

Figure 1a reports the distribution of percentage variation of ED visits in period 2 compared with the mean number of visits in period 1 for each participating centre, which showed a consistent and significant trend for a decrease in ED accesses in period 2. That happened for UTI diagnoses in most centres (Milan and Brescia were the only exceptions), as indicated in Fig. 1b. In contrast, a significant trend for positive percentage variation of diagnosis rate was observed in most centres (12 out of 17), as shown in Fig. 1c.

**Fig. 2:**
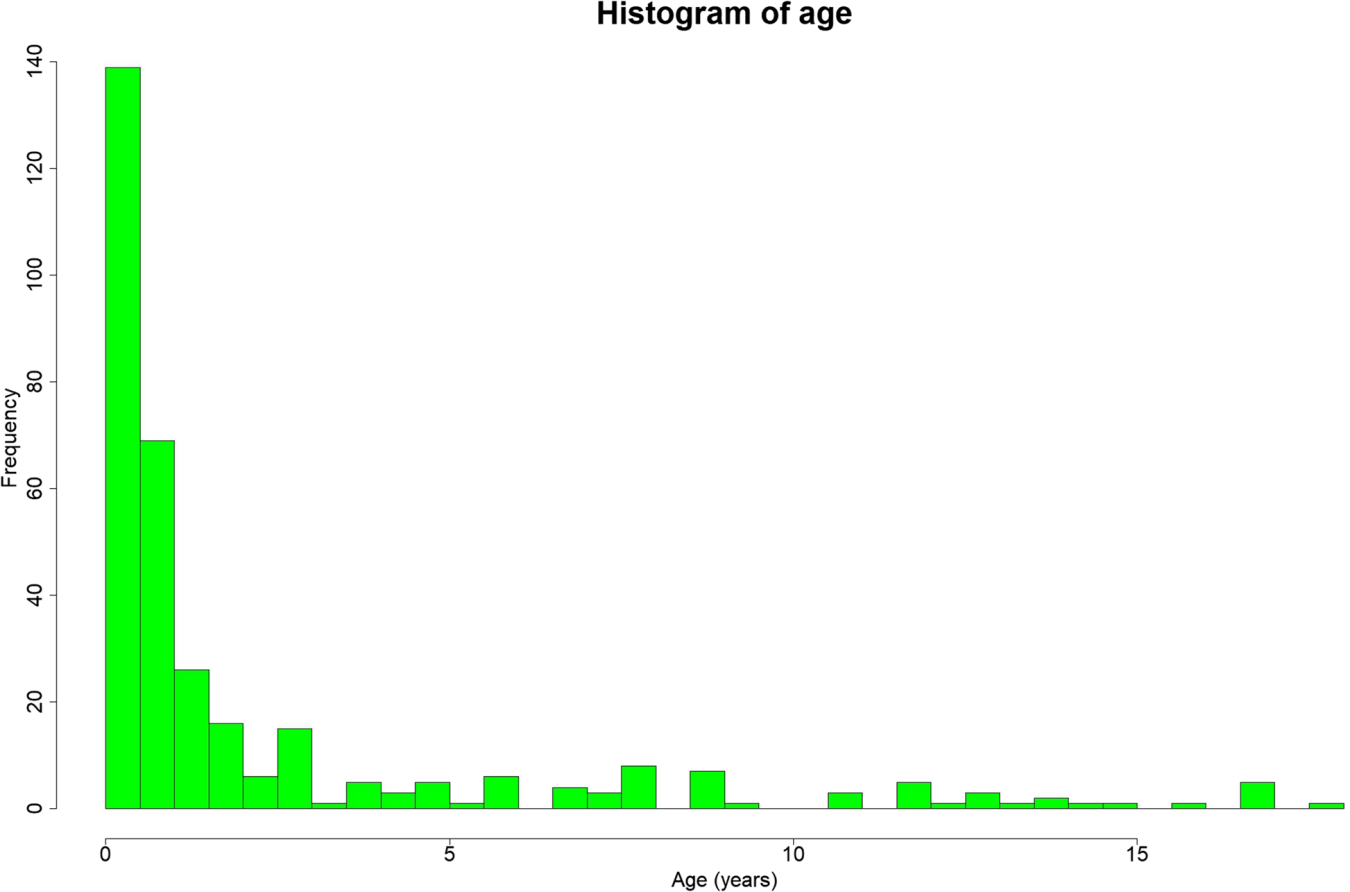
Histogram of age distribution of patients with diagnosis of febrile UTI. The distribution is absolutely not normal, as shown also by a Shapiro Wilk test (*p* value < 0.001)

Distribution of age in patients diagnosed with febrile UTI was right skewed and absolutely not normal, with a very high frequency in patients in the first year, as shown in the histogram in Fig. 2.

No significant differences were noted in period 2 for age (Table 3), gender, access mode, history of previous UTI, and urinary tract malformations compared with mean values in period 1 (Table 2).

In period 2 there was a lower rate of cases admitted with a one-day history of fever and an increase in patients with two or > 2 days of fever (*p* = 0.02). A significant increase in hospitalization rate was also documented: 63% vs 45.5%, *p* = 0.008 (Table 4). There were no significant differences in days of hospitalization and days of fever after admission (*p* = 0.58 and *p* = 0.34, respectively).

**Table 4:** Children with UTI diagnosis: clinical features

	Period 1	Period 2	*p* value
Days of fever before ED visit			
1	62%	47%	0.02
2	18%	23%	
> 2	20%	30%	
Hospitalization	45.5%	63%	0.008
Type of germ In urine culture			
E. coli	82.7%	74%	0.02
P. aeruginosa	0.8%	6,8%	
E. faecalis	2.3%	5.5%	
Klebsiella pn	3.8%	4.1%	
Proteus	4.1%	2,7%	
Other	6.4%	6.8%	

As reported in Table 3, laboratory data collected after admission to the ED showed no significant differences for WBC and neutrophils (N) count. At the same time, CRP values were significantly higher in period 2 vs period 1 (Table 3). Analysis of the type of bacterium found in urine cultures revealed a significantly higher percentage *Pseudomonas aeurginosa* and *Enterococcus faecalis* species and reduction in *E. coli* isolation in period 2 compared to period 1 (*p* = 0.02, Table 4).

Ninety-three blood cultures were collected during the 3-year study; however, only 11 (11.8%) were positive. Positive blood cultures, in percentage, were comparable between 2020 and the previous two years: 3/24 (12.5%) in 2020 vs 8/69 (11.6%) in 2018 + 2019, *p* = 0.9. E. coli was found in 6/8 (75%) blood cultures in 2018–2019 and in 1/3 (33.3%) blood cultures in 2020. No statistically significant differences were observed in the distribution of bacteria at blood cultures in 2018–2019 compared to 2020.

Regarding routes of antibiotic therapy administration, a significantly larger use of intravenous antibiotics was detected in 2020 compared to 2018 and 2019 (57.1% vs 42.1%, *p* = 0.025).

## Discussion

The data presented in the current study highlighted a reduction in febrile UTI diagnoses and an even significant decline in children’s ED evaluations during the first Covid-19 pandemic compared to data referring to the previous two years. Furthermore, febrile UTIs during the study period shared characteristics of greater severity than in the same months of 2018 and 2019.

Our study’s centres were located in Northern Italy, the area most affected during the first wave of the Covid-19 pandemic. Between March and April 2020, there was a remarkable reduction in pediatric ED activity compared to the previous two years (-75.1%). Consequently, the rate of UTI diagnoses showed a statistically significant increase in 2020.

These observations were in line with what has already been reported by other authors. Vierucci et al. found a reduction of -78.9% in pediatric ED visits in March 2020 vs February 2020 in a general ED in Lucca, Tuscany, central Italy [13]. An American study including 144 ED and four urgent care centres stated that pediatric ED accesses declined more markedly than adult non-Covid-19 visits during the period January-June 2020 vs the same period in 2019 (-74% of children under ten years and -67% of 14–17-year-olds vs -60% of adult visits) [15].

These trends could be explained by a general reduction in traumatic injuries and respiratory and gastrointestinal infections in children during the lockdown period [13, 15, 21]. Several studies also reported that parents were worried about hospital-acquired Sars-Cov-2 infection [13, 14]. However, according to an European multicenter study, children and adolescents had a much lower risk of contracting the virus, and severe forms were exceptional in this population [22]. Indeed, these data differ from those observed in developing countries where children and adolescents hospitalized with Covid-19 have higher morbidity and mortality rates of multifactorial origin, including social and biological factors [23, 24]. The fear of the unknown and confusing information on social media regarding hospital emergency care led parents to consider hospitals no longer safe for their children. Consequently, parents preferred to keep their children at home with either deferrable health problems or life-threatening conditions, such as acute leukaemia, cerebral and abdominal masses, acute perforated appendicitis, and severe diabetic ketoacidosis [14, 25–28].

Our study is the first to report the effect of the first Covid-19 pandemic on febrile UTIs diagnosed in children in a pediatric ED setting. In 15 out of 17 centres, we observed a significant reduction in febrile UTI diagnoses throughout the pandemic compared with the previous two years. However, in proportion, the reduction in ED visits for UTI was less than for all other ED accesses (45.1% vs 75.1%). This occurrence can be explained by UTI symptoms which can be severe, such as malaise, abdominal pain, and long-lasting fever, especially in cases of antibiotic treatment delay. High fever, in particular, has always been a source of great concern for parents. Fear that the child’s medical condition may rapidly worsen led parents to overcome, at least in some cases, their fear of the risk of contracting Sars-Cov-2 while waiting for the pediatric visit in the ED. It is also plausible that febrile children were brought to the ED because of their parents’ fear of having a Sars-Cov-2 infection.

On the other hand, we collected data indicating a delay in diagnosing febrile UTI in the ED. The study showed a more extended period of fever before accessing the ED, a higher hospitalization rate, and a greater tendency to use intravenous antibiotics. Moreover, the children brought to the ED were more severely ill. Significantly higher CRP values in period 2 vs period 1 supported this claim. Since several studies found that CRP values at admission in clinically diagnosed acute pyelonephritis were higher in patients with scars on ^99m^Tc-dimercaptosuccinic acid (DMSA) scintigraphy [29, 30], we could hypothesize that the risk of renal damage would be increased in this patients sample. We supposed that the reason for more server illnesses diagnosed in ED during 2020 is partly due to parents’ fear of contracting Sars-Cov-2 infection in healthcare settings. This fear could probably explain why children with suspected UTI were brought late to the attention of the ED, remaining at home for several days since fever started, as we previously demonstrated. Another reason, closely related to the previous one, could be the delay in starting the specific antibiotic treatment based on the urine culture’s outcome and the antibiogram.Furthermore, since ED UTI diagnoses were reduced in period 2 compared to period 1 in almost all participating centres, we speculated that many children with febrile UTIs were managed at home. This group has likely been treated empirically with antibiotics, probably without a formalized diagnosis after several days of fever. It represented another group of pediatric patients probably hard to characterize in the future and with the possible progression of kidney damage.

Our findings concerning the urine suggested a difference in the germs distribution in period 2 compared to period 1. We found an increased prevalence of atypical germs and reduced isolation of *E. coli* in urine specimens collected in children with febrile UTI accessing the ED during the first Covid-19 pandemic compared to the results of urine cultures in period 1. We hypothesized that this data is attributable to the fact that during 2020 UTIs showed higher severity, and isolation of atypical germ at urine culture is known to be a risk factor for the worst outcome with parenchymal damage [31]. Another hypothesis could be that more children with malformative uropathy, usually more prone to infections due to bacteria other than *E. coli*, accessed the ED than in the previous two years.

This study has some limitations. Although it is a multicenter retrospective study, even if the initial target was to sample throughout Italy, all the data were collected from EDs in the country’s north. This condition can also be a strength since we focused on pediatric febrile UTIs in the worst Covid-19 affected area. and learned how to manage it.

## Conclusions

In conclusion, we found a significant increase in febrile UTI diagnoses in 2020 (period March–April) compared to 2018 and 2019 due to a more significant reduction of ED visits than UTI diagnoses. The first Covid-19 pandemic delayed access of febrile UTIs to EDs, resulting in higher hospitalization rates, greater tendency to use intravenous antibiotics, and more elevated CRP values, thus causing more severe disease that deserves careful follow-up in the future.

## Data Availability

All data described in the manuscript, including all relevant raw data, are available. To access the data is necessary to consult the corresponding author Hospital Ethical Commetee. Data were collected in a database built ad hoc for the purposes of the study. Please contact dr Laura Cesca to have access to study’s data.

## Abbreviations

ED: Emergency department
UTI: Urinary tract infection
CPR: C reactive protein
COVID19: Coronavirus disease 19
Sars-Cov-2: Severe Acute Respiratory Syndrome Coronavirus 2
CAKUT: Congenital anomalies of the kidney and urinary tract
SINEPE: Italian Society of Pediatric Nephrology
WBC: White blood cell count
DMSA: Dimercaptosuccinic acid
N: Neutrophils
